# E47 as a novel glucocorticoid-dependent gene mediating lipid metabolism in patients with endogenous glucocorticoid excess

**DOI:** 10.3389/fendo.2023.1249863

**Published:** 2023-11-17

**Authors:** Wei Zhang, Hanna Nowotny, Marily Theodoropoulou, Julia Simon, Charlotte M. Hemmer, Martin Bidlingmaier, Matthias K. Auer, Martin Reincke, Henriette Uhlenhaut, Nicole Reisch

**Affiliations:** ^1^ Medizinische Klinik and Poliklinik IV, Klinikum der Universität München, Ludwig-Maximilians-Universität (LMU) München, Munich, Germany; ^2^ Molecular Endocrinology, Institutes for Diabetes and Obesity & Diabetes and Cancer IDO & IDC, Helmholtz Zentrum Muenchen (HMGU) and German Center for Diabetes Research (DZD), Munich, Germany; ^3^ Metabolic Programming, Technische Universität München (TUM) School of Life Sciences Weihenstephan and ZIEL Institute for Food & Health, Munich, Germany

**Keywords:** Cushing’s syndrome, Cushing’s disease, transcription factor 3, comorbidity, dyslipidemia, ACTH, cortisol

## Abstract

**Purpose:**

E47 has been identified as a modulating transcription factor of glucocorticoid receptor target genes, its loss protecting mice from metabolic adverse effects of glucocorticoids. We aimed to analyze the role of E47 in patients with endogenous glucocorticoid excess [Cushing’s syndrome (CS)] and its association with disorders of lipid and glucose metabolism.

**Methods:**

This is a prospective cohort study including 120 female patients with CS (ACTH-dependent = 79; ACTH-independent = 41) and 26 healthy female controls. Morning whole blood samples after an overnight fast were used to determine E47 mRNA expression levels in patients with overt CS before and 6–12 months after curative surgery. Expression levels were correlated with the clinical phenotype of the patients. Control subjects underwent ACTH stimulation tests and dexamethasone suppression tests to analyze short-term regulation of E47.

**Results:**

E47 gene expression showed significant differences in patient cohorts with overt CS vs. patients in remission (*p* = 0.0474) and in direct intraindividual comparisons pre- vs. post-surgery (*p* = 0.0353). ACTH stimulation of controls resulted in a significant decrease of E47 mRNA expression 30 min after i.v. injection compared to baseline measurements. Administration of 1 mg of dexamethasone overnight in controls did not change E47 mRNA expression. E47 gene expression showed a positive correlation with total serum cholesterol (*p* = 0.0036), low-density lipoprotein cholesterol (*p* = 0.0157), and waist–arm ratio (*p* = 0.0138) in patients with CS in remission.

**Conclusion:**

E47 is a GC-dependent gene that is upregulated in GC excess potentially aiming at reducing metabolic glucocorticoid side effects such as dyslipidemia.

## Introduction

Chronic cortisol excess leads to a substantially increased cardiovascular risk and overall mortality (1). Patients with Cushing’s syndrome (CS) suffer from multiple glucocorticoid (GC) excess-related alterations of protein, lipid, and carbohydrate metabolism resembling those found in metabolic syndrome (2). Overt diabetes mellitus has been described in about a third of patients with CS of adrenal and pituitary origin and even in 74% in patients with ectopic CS (3); impaired glucose tolerance is equally frequent (4). The main underlying mechanisms are induction of gluconeogenesis, disruption of insulin receptor signaling, and pancreatic β-cell dysfunction by GC. Dyslipidemia is another prominent feature of CS with increased circulating very-low-density lipoprotein (VLDL) and LDL, but not high-density lipoprotein (HDL), leading to an elevation of triglycerides and total cholesterol concentrations (5). GCs enhance lipolysis in peripheral fat depots, promote pre-adipocyte differentiation in central fat, and modulate free fatty acid mobilization. A high prevalence of hepatic steatosis was also reported in CS correlating with total abdominal and visceral fat (6). The resulting changes in body composition with accumulation of central adipose tissue as well as GC-induced proximal muscle atrophy are typical clinical characteristics of CS.

The effects of GCs are mainly mediated through the glucocorticoid receptor (GR). Glucocorticoid receptors (GR) are ubiquitously expressed and composed of an N-terminal transactivation domain, the central DNA-binding domain, and the C-terminal ligand-binding domain (7, 8). Binding of cortisol initiates translocation of the ligand–receptor complex to the nucleus, where it can bind to glucocorticoid response elements (GREs) or different transcription factors such as activator protein-1 (AP-1) and NF-κB (7), leading to induction or repression of the transcription of target genes. Biological responses to GCs therefore vary substantially across different tissues (8) as demonstrated by the various diverse functions that are exhibited by GCs. Processes involved in this complex and dynamic regulation of transcription by GR include variation in GR binding sites, differences in chromatin accessibility, and presence of and interaction with other transcription factors or transcriptional coregulators (9–13).

One of these associated transcription factors is E47, also known as transcription factor 3 (TCF3). E47 has been first described as a required factor for B-cell development (14), promoting commitment to the B-cell lineage and B-cell maturation (15). Moreover, E47 regulates the expression of genes involved in cell survival, cell cycle progression, lipid metabolism, and stress response (16). E47 is an E-box binding protein, and recently, enrichment in E-boxes specifically near GRE in hepatic promoters and enhancers has been reported (17). E47 modulates GR-dependent gene activation in mice, specifically in hepatocytes influencing hepatic lipid and glucose metabolism. Interestingly, in mice, loss of E47 impaired the upregulation of metabolic target genes and mutant mice were protected from steroid-induced hyperglycemia, dyslipidemia, and hepatic steatosis (17). A high-throughput luciferase reporter screen of *cis*-regulatory sequences that are supposed to be regulated by GR confirmed that human GR targets are also co-regulated by E47 (17).

Consequently, the aim of this study was to investigate the role of E47 in human disease. We hypothesized that the expression of E47 levels is associated with metabolic comorbidities in patients with CS. Intrigued by the findings in mice, we were particularly interested in examining the potential link between E47 expression and alterations in glucose and lipid metabolism in patients with endogenous CS.

## Subjects and methods

### Subjects

Subjects of this study were prospectively recruited from the Department of Medicine IV of the University Hospital Munich, Germany. All patients provided written informed consent to participate in the German Cushing’s Registry (NeoExNET, ethical approval no. 152-10). A total of 120 female patients with CS were included in our analysis. The majority of subjects had ACTH-dependent CS, 68 of them due to excess secretion of ACTH by a benign pituitary adenoma (central CS) and 11 patients were due to ectopic ACTH secretion (ectopic CS). The remaining 41 patients had ACTH-independent CS. Additionally, 26 healthy female subjects matched for BMI were recruited as controls. For data analysis, patients with CS were further subdivided into overt CS with persistent cortisol excess and those in remission. Diagnostic criteria applied to the diagnostic approach outlined by the evidence-based 2008 Endocrine Society clinical guidelines (18–20). All patients were prospectively enrolled in the German Cushing Registry. Prior to biochemical evaluation, we documented the presence or absence of characteristic clinical signs and symptoms as well as comorbidities typical for CS. We used recommended standard biochemical testing including the low-dose dexamethasone suppression test (LDDST), late-night salivary cortisol (LNSC), and urinary free cortisol (UFC) in a 24-h collection using validated assays as described previously (21, 22). After clinical and biochemical confirmation of CS, we determined the origin of CS with measurement of ACTH, pituitary MRI imaging, CRH test, and, if needed, inferior sinus petrosus sampling. The diagnosis of CS was confirmed by surgery (histology, adrenal insufficiency after surgery, and clinical improvement). We defined successful surgery leading to remission as a phase of adrenal insufficiency post-surgery, requiring glucocorticoid replacement therapy, and a regression of clinical symptoms (23). Patients were examined 1, 3, 6, and 12 months after surgery and annually thereafter (checklist-based clinical examination, recording of new or worsening comorbidities combined with biochemical testing by 1-mg LDDST, UFC, and LNSC). Depending on the clinical and biochemical results, we classified patients as being in remission or as having suspected recurrence. Exclusion criteria included intake of glucocorticoid or ACTH-related drugs.

### Sample collection

Of each study participant, 2.7 mL of venous blood was drawn using EDTA Monovettes (Sarstedt, Germany). The blood samples were directly placed on ice. Blood and DL buffer (NucleoSpin RNA Blood *Midi* kit, MACHEREY-NAGEL, Düren, Germany) containing guanidine thiocyanate were added in a 1:1 ratio and immediately frozen at −80°C. Frozen samples were subsequently used for RNA extraction.

For ACTH stimulation tests, healthy controls were asked to remain in a lying position. A peripheral line was placed; after 15 min, blood was drawn at baseline, then 250 µg Synacthen (Alfasigma, Italy) was administered intravenously and 30 minutes later blood was drawn for the stumlated sample.

For dexamethasone suppression test, controls were administered 1 mg of dexamethasone (Mibe, Germany) at 11 p.m. and serum cortisol was measured at 8 a.m. the following morning.

### RNA isolation

RNA was isolated from 1 mL of whole blood using NucleoSpin RNA Blood *Midi* kit, (MACHEREY-NAGEL, Düren, Germany) according to the manufacturer’s protocol. RNA samples were quantified by a NanoDrop Spectrophotometer (NanoDrop 1000, Thermo Fisher Scientific) and were stored at −80°C for subsequent use. RNA purity was determined by the ratio of absorbance at 260 and 280 nm and defined as A260/280 > 1.8.

### Quantitative real-time RT-PCR

The SuperScript II cDNA synthesis kit (Invitrogen, Carlsbad, CA, USA) was used for reverse transcription of 100 ng of RNA following the company’s instruction. The primer pairs used were as follows (Eurofins Genomics, Germany):

E47 forward 5′-CCTGAACTTGGAGCAGCAAG-3′

E47 reverse 5′-TACCTTTCACATGTGCCCGG-3′

β-actin forward 5′-CATGTACGTTGCTATCCAGGC-3′

β-actin reverse 5′-TGAGGATCTTCATGAGGTAG-3′

Quantitative real-time polymerase chain reaction (RT-qPCR) was performed in the Quantstudio 5 real-time PCR systems (Applied Biosystems). PCR reactions were carried out in a 10-μL reaction mixture. For each PCR reaction, 5 μL (5 ng) of template cDNA, 4 μL of SsoFast EvaGreenSupermix (Bio-Rad Laboratories Inc, USA), and 0.5 μL (5 pmol) of each primer were used. The PCR cycling conditions were as follows: an initial denaturation step (95°C, 10 min), followed by 40 cycles of denaturation (95°C, 15 s) and annealing/elongation (60°C, 60 s).

Data were obtained as Ct, and ΔCt = (Ct of E47) − (Ct of β-actin) was calculated. Normalized E47 expression was calculated using the 2^−ΔΔCt^ method (ΔΔCt = (ΔCt of CS) − (median ΔCt of controls)). If no control data for normalization were available, data were normalized to the median ΔCt of the enrolled control group.

### Statistical analysis

Results are expressed as median with 95% CI. *p* < 0.05 was considered statistically significant. For PCR analysis, data were tested for statistical significance using normalized E47. To test for normality, Shapiro–Wilk’s test was used. As values of E47 mRNA expression were non-normally distributed, Kruskal–Wallis test and Dunn’s multiple comparisons test were used for comparison of E47 expression between overt CS, remission CS, and the control group. Intraindividual change of E47 expression pre- vs. post-surgery as well as the two described endocrine function tests were analyzed using Wilcoxon matched-pairs signed rank test. Comparisons between different CS entities were analyzed by Mann–Whitney test. To determine if potential differences in E47 expression between groups were independent of differences in age, we additionally performed a mixed model analysis with group and age as fixed effects and subject as a random effect. Bivariate correlations between variables were performed using nonparametric Spearman’s correlation. GraphPad Prism 8 (GraphPad Software, San Diego, CA, USA) was used for statistical analysis and graphical presentation of obtained data sets.

## Results

### Characteristics of study participants

Of the 120 female patients with CS included in our analysis, 102 patients underwent surgery. Median age was 54 years (IQR 41–63 years) in the overt group (*n* = 29) and 52.0 years (IQR 42–63 years) for patients in remission (*n* = 91). Median age of the healthy control cohort (*n* = 26) was 31.5 years (IQR 26–36 years), which was significantly lower than in the two subgroups of patients with CS (*p* < 0.0001). **Table 1** displays the characteristics of participants, including numbers, age, 24-h-urine cortisol levels, and investigated metabolic parameters [weight, body fat mass, waist–arm ratio, waist–hip ratio, blood pressure, glucose, insulin, homeostatic model assessment-insulin resistance (HOMA), HbA1c, cholesterol, triglycerides, HDL, and LDL]. Glucose concentrations (*p* = 0.0037), 24-h-urine cortisol (*p* < 0.0001), systolic blood pressure (*p* = 0.0326), insulin (*p* = 0.0086), and HOMA (*p* = 0.0050) in overt CS were higher than in patients with CS in remission and the control group.

**Table 1:** Subject characteristics.

	overt CS				remission CS				control		"P-value	$P-value
	ACTH-dependent CS		ACTH-independent CS		ACTH-dependent CS		ACTH-independent CS					
	N	Mdn-IQR	N	Mdn-IQR	N	Mdn-IQR	N	Mdn-IQR	N	Mdn-IQR		
*Age, yrs	16	45 (33.5-57)	13	59 (53-68)	63	54.5 (45-63)	28	48 (38.5-64.5)	26	31.5(26-36)	0.9310	0.5800
Body composition												
Weight, kg	16	80 (65-94)	13	80 (74-90)	63	76 (62-87)	28	76 (61-84)	25	89 (71-117)	0.0710	0.9500
BMI, kg/m^2^	15	30.48 (25.50-34.52)	13	30.78 (29.20-34.96)	62	29.18 (24.09-34.07)	26	27.18 (22.92-31.05)	26	32.13 (26.47-40.62)	0.0865	0.3162
Body fat mass, %	16	36.3 (33.6-45)	13	34.7 (24.5-42.3)	63	31.4 (26.1-38)	28	35.8 (30.2-38.5)	15	32.8 (45.5-8.7)	0.0970	0.8320
Waist-arm-ratio	16	3.13 (2.86-3.6)	13	3.06 (2.93-3.27)	63	3.13 (2.83-3.38)	28	2.81 (2.72-3.07)	26	2.96 (2.65-3.24)	0.3630	0.0220
Waist-hip-ratio	16	0.86 (0.81-0.95)	13	0.89 (0.81-0.92)	63	0.87 (0.79-0.94)	28	0.82 (0.78-0.92)	26	0.87 (0.75-0.93)	0.8810	0.2370
Blood pressure												
*RR-sys, mmHg	16	129.33 (117.33-139)	13	127 (118-146)	63	121 (111.67-133)	28	122 (110-132)	25	117.33 (108.33-129.33)	0.0326	0.8620
RR-dia, mmHg	16	82 (77-87)	13	80 (74-82.7)	63	79 (72.67-83)	28	79.2 (72-84.3)	25	77.67 (73-83)	0.1110	0.7590
Serum biochemistry												
*Glucose, mg/dl	16	92.5 (88.5-95)	13	106 (97-121)	63	89.5 (85-99)	28	88 (84-101)	24	99.5 (87.5-102)	0.0037	0.3220
HbA1c, %	16	5.7 (5.3-6.1)	13	5.8 (5.4-6.6)	63	5.5 (5.2-5.8)	28	5.45 (5.2-5.9)	25	5.3 (5.1-5.7)	0.0740	0.8380
Cholesterin, mg/dl	16	208 (174-222)	13	198 (179-215)	63	204 (178-231)	28	192 (177-210)	24	206.5 (188-226)	0.8660	0.2080
TG, mg/dl	16	111 (72-178)	13	119 (104-165)	63	125 (83-180)	28	125 (94-165)	24	126 (72.5-210.5)	0.8970	0.6700
HDL, mg/dl	16	71 (54-75)	13	56 (50-63)	63	63 (48-75)	28	57 (48.5-69)	24	52 (41-69.5)	0.7170	0.1850
LDL, mg/dl	16	102 (87-140)	13	107 (96-138)	63	121 (89-140)	28	106 (88.5-123)	22	124.5 (101-132)	0.8610	0.3300
*24hUFC, ug/24h	16	170 (96-302)	13	158 (81.3-218)	63	37.5 (9-58)	28	43.3 (8-62)	25	93 (62 115)	< 0.0001	0.2750
*Insulin, pmol/L	16	14.4 (5.9-22)	13	11.1 (10-19.8)	63	8.8 (5.5-14.6)	28	8.9 (4.7-14)	24	15.8 (9.1-24.5)	0.0086	0.9380
*HOMA-IR	16	3.47 (1.3-5.2)	13	3.05 (2.85-5.18)	63	2.12 (1.19-3.35)	28	2.07 (1-3.53)	24	3.93 (1.99-6.19)	0.0050	0.7630

n, number; Mdn, median; IQR, interquartile range; RR-sys, blood pressure systolic; RR-dia, blood pressure diastolic; HbA1c, glycated hemoglobin; TG, triglyceride; HDL, high-density lipoprotein; LDL, low-density lipoprotein; 24h-UFC, 24-hour urinary free cortisol; HOMA-IR, homeostatic model assessment–insulin resistance. #: p-value between the overt group and the remission group, $: p-value between the ACTH-dependent CS subgroup and the ACTH-independent CS subgroup, p-value < 0.05 (*).

### E47 gene expression

**Figure 1:**
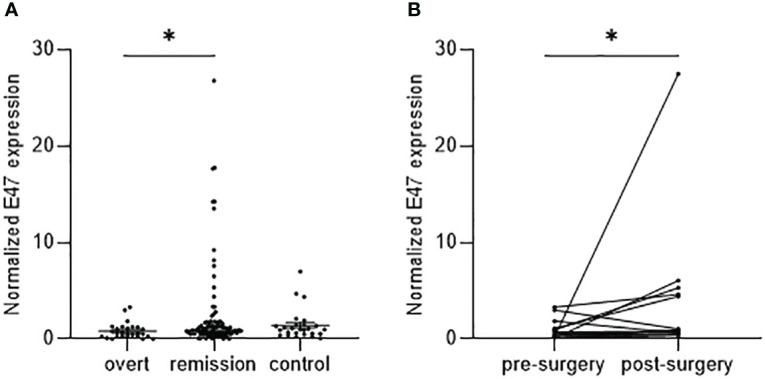
E47 gene expression in patients with CS correlates with disease status. **(A)** Normalized E47 transcript levels in whole blood samples of patients with CS in remission (*n* = 91) are increased compared to overt CS (*n* = 29), while no difference can be observed compared to control subjects (*n* = 26). Data are shown as median with 95% CI; *p*-value <0.05 (*) by Kruskal–Wallis test with Dunn’s multiple comparison test. **(B)** E47 expression is increased in the same individual patients post-surgery (*n* = 14); *p*-value <0.05 (*) by Wilcoxon matched-pairs signed rank test (*p* = 0.0353).

To determine cortisol-dependent differences of E47 gene expression, we compared E47 mRNA gene expression in 120 patients with CS and 26 healthy controls with physiological baseline cortisol levels. E47 gene expression in whole blood samples was significantly lower in patients with overt CS (*n* = 29) compared to patients in remission (*n* = 91; *p* = 0.0474). No statistical difference was observed between E47 expression in patients with CS in remission and healthy controls (**Figure 1A**). The differences between patients in remission in comparison to those with overt CS remained significant after controlling for age in mixed model analysis (*F*_1,146 = _3.140; *p* = 0.046).

In direct intraindividual comparisons of E47, mRNA expression was significantly lower in the overt Cushing status pre-surgery compared to matched samples of the individual patients in remission post-surgery (*n* = 14; *p* = 0.0353) (**Figure 1B**).

**Figure 2:**
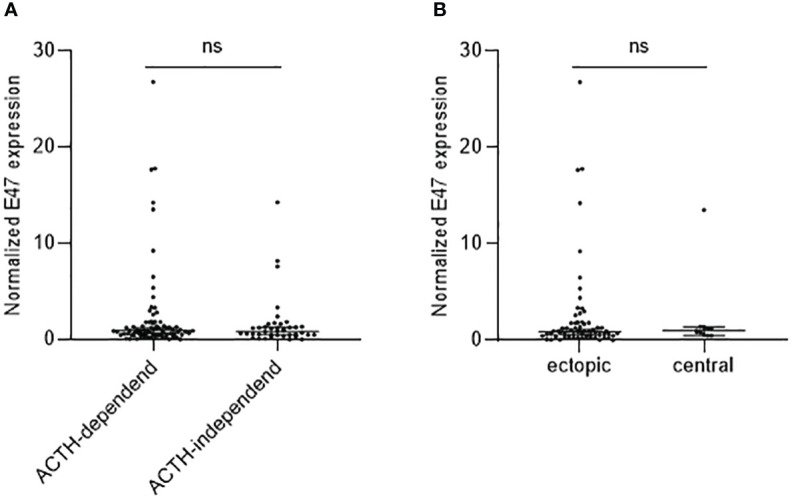
E47 mRNA expression in ACTH-dependent and -independent CS. E47 expression in patients with ACTH-dependent (*n* = 79) and ACTH-independent (*n* = 40) CS **(A)** as well as in patients with ectopic (*n* = 68) or central (*n* = 11) CS **(B)** is not significantly different as determined by Mann–Whitney test.

We analyzed E47 expression according to whether CS was ACTH-dependent (*n* = 79) or -independent (*n* = 40). No differences in E47 gene expression were observed in ACTH-dependent vs. -independent overt CS (**Figure 2A**). We furthermore analyzed within the group of patients with ACTH-dependent CS whether normalized E47 expression is altered. No statistically significant differences between patients with ectopic CS (*n* = 68) and those with central CS (*n* = 11) could be observed (**Figure 2B**).

**Figure 3:**
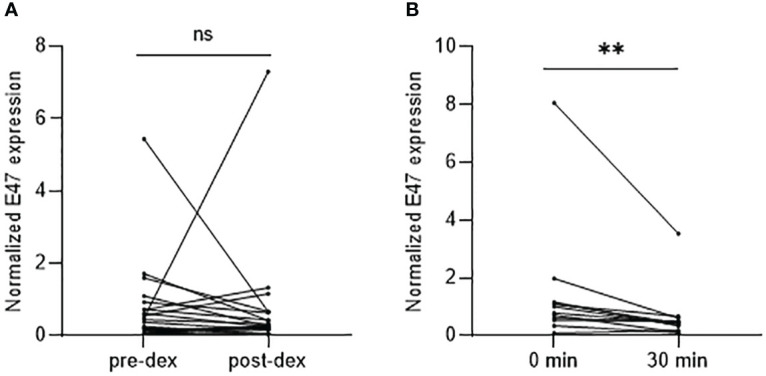
E47 mRNA expression after dexamethasone suppression test and ACTH stimulation test. **(A)** E47 mRNA expression showed no statistical difference pre- or post-administration of 1 mg of dexamethasone (*n* = 23). **(B)** E47 mRNA expression was significantly reduced 30 min after i.v. injection of Synacthen^®^ (*n* = 10); *p*-value < 0.05 (*) by Wilcoxon matched-pairs signed rank test (*p* = 0.0015). ns, not significant.

To further understand the dynamics of E47 gene expression following glucocorticoid exposure, we performed dexamethasone suppression test and ACTH stimulation test on healthy controls. Administration of 1 mg of dexamethasone (*n* = 23) did not significantly change E47 mRNA levels (**Figure 3A**). In contrast, ACTH administration (*n* = 12) significantly decreased E47 expression 30 min after i.v. injection compared to baseline measurements (*p* = 0.0015) (**Figure 3B**).

### Correlation of E47 gene expression with clinical parameters

**Figure 4:**
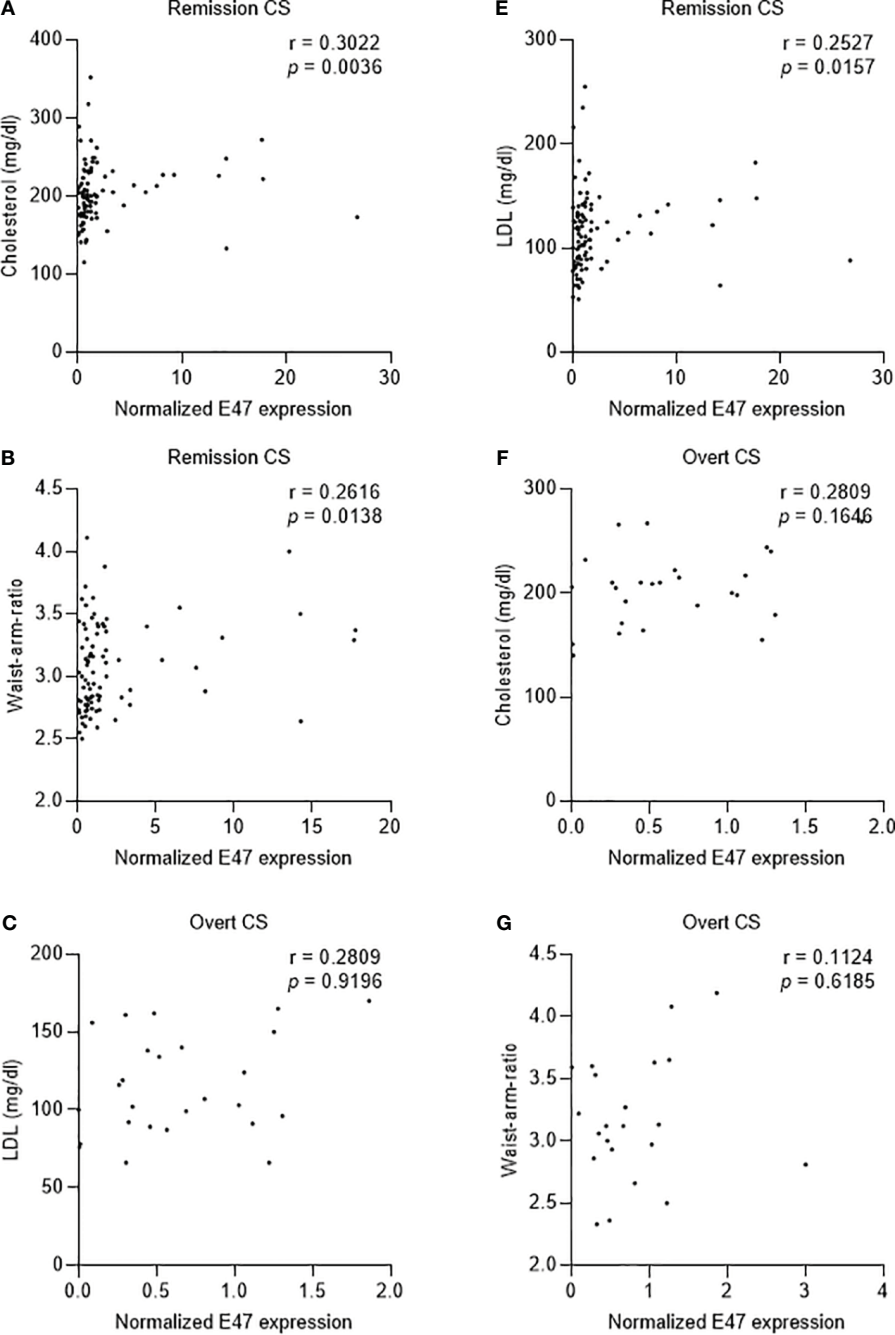
Correlation of E47 gene expression and different clinical parameters in patients with overt CS and CS in remission. **(A–C)** E47 gene expression shows positive correlation with total serum cholesterol (*p* = 0.0036, *n* = 90, *r* = 0.3022), serum LDL (*p* = 0.0157, *n* = 90, *r* = 0.2527), and waist–arm ratio (*p* = 0.0138, *n* = 88, *r* = 0.2616). **(D–F)** E47 gene expression shows no significant correlation with total serum cholesterol (*p* = 0.1646, *n* = 27, *r* = 0.2809), serum LDL (*p* = 0.9196, *n* = 27, *r* = 0.209), and waist–arm ratio (*p* = 0.6185, *n* = 23, *r* = 0.1124). *p*-value < 0.05 is considered significant as determined by nonparametric Spearman correlation.

We further examined the correlation between clinical parameters and E47 mRNA expression. In patients with CS in remission (total *n* = 90), E47 gene expression showed only a positive correlation with total serum cholesterol (*p* = 0.0036, *r* = 0.3022), LDL (*p* = 0.0157, *r* = 0.2527), and waist–arm ratio (*p* = 0.0138, *r* = 0.2616) (**Table 2**; **Figures 4A–C**). No correlations were found in patients with overt CS (total *n* = 27) (**Table 2**; **Figures 4D–F**).

**Table 2:** Correlations of E47 gene expression with clinical parameters.

Group			Age (year)	BMI (kg/m^2^)	Body fat (%)	WHR	LDDST (µg/l)	UFC (µg/24h)	LNSC (µg/l)	Hb1c (%)	HOMA- IR	TG (mg/dl)	Total cholesterol (mg/dl)	HDL (mg/dl)	LDL (mg/dl)	RR-sys (mmHg)	RR-dia (mmHg)
Overt CS	E47	ρ	.058	-.083	-.033	.090	-.053	.082	-.069	.179	.089	-.120	-.208	.060	-.137	.112	-.123
		P	.764	.674	.915	.685	.796	.685	.728	.362	.658	.550	.297	.767	.494	.570	.531
		N	29	28	13	23	26	27	28	28	27	27	27	27	27	28	28
Remission	E47	ρ	-.031	-.014	.044	-.188	.095	-.136	.001	-.055	-.063	-.105	-.306"	-.052	-.257	-.016	-.119
		P	.769	.894	.766	.079	.625	.255	.992	.604	.563	.324	.003	.624	.015	.883	.271
		N	90	89	48	88	29	72	67	90	86	90	90	90	90	87	87
Controls	E47	ρ	.027	.141	-.077	-.042	.121	.267	-.181	-274	.157	.024	-.094	.163	-.172	.246	.328
		p	.897	.502	.785	.846	.583	.196	.386	.186	.465	.910	.662	.446	.443	.236	.110
		N	26	25	15	24	23	25	25	25	24	24	24	24	22	25	25

ρ: Spearman’s rank correlation coefficient; CS: Cushing’s syndrome; WHR: waist-to-hip ratio; LDDST: low-dose dexamethasone suppression test; UFC: urinary free cortisol; LNSC: late-night salivary cortisol; HOMA-IR: Homeostasis Model Assessment of Insulin Resistance; TG: triglycerides; RR-sys: systolic blood pressure; RR-dia: diastolic blood pressure.

## Discussion

This is the first study to investigate the role of E47 in a human disease model of endogenous GC excess. In an attempt to translate the findings from mice to humans, we investigated the expression and correlation of E47 with metabolic adverse effects in patients with endogenous CS.

We found that E47 gene expression is significantly lower in patients with overt CS than in patients in remission. When comparing E47 mRNA expression directly intraindividually pre- (overt CS) and post-surgery in remission, this difference in E47 gene expression became even clearer. These findings indicate that E47 expression is significantly downregulated in case of excessive exposure to glucocorticoids. To confirm this GC-dependent downregulation of E47 and the dynamics of E47 in humans, we exposed healthy controls to a short Synacthen^®^ test. Upon ACTH stimulation, E47 mRNA expression was significantly downregulated within 30 min compared to basal mRNA expression. These results highlight E47 as a glucocorticoid-dependent gene. We, however, did not observe differences in E47 gene expression upon administration of 1 mg of dexamethasone, nor did we see a direct correlation between E47 gene expression and 24hUFC. Non-significant changes in E47 expression after administration of 1 mg of dexamethasone may be due to insufficient overall change in glucocorticoid exposure of the GR compared to a physiologic state of glucocorticoids not causing prominent changes in E47 gene expression. While endogenous GC are downregulated in the dexamethasone suppression test, dexamethasone as exogenous GC may counter-regulate this effect. Another hypothesis is that the time point of change in E47 expression was solely missed. Furthermore, no direct correlation was seen between 24hUFC, baseline cortisol concentrations or AUC of saliva cortisol, and peripheral blood E47 mRNA expression. Therefore, how E47 gene expression is specifically regulated in glucocorticoid excess states needs to be further investigated. One may only speculate that there is no linear correlation with glucocorticoid concentration but a ceiling effect may occur.

E47 knockout mutant mice showed an improved metabolic phenotype in response to GC treatment; in particular, these mice showed lower glucose concentrations and less lipid accumulation in the liver and circulation, and did not develop hepatic steatosis (17). We therefore hypothesized that the observed downregulation of E47 in overt CS compared to patients in remission and the rapid, inducible downregulation of E47 upon stimulation of ACTH aims to mitigate the GC-induced metabolic adverse effects as well in humans.

Consistent with the findings in mice, we found that patients with lower E47 expression showed lower total cholesterol concentrations and lower LDL concentrations than those with higher E47 expression. Based on the findings in mice, it would also have been interesting to have detailed liver imaging to evaluate and correlate E47 with the degree of potential hepatic steatosis. Owing to the lack of availability of these data, a potential correlation could not be analyzed. Hemmer et al. could previously show *in vivo* that E47 knockout mice could be protected from steroid-related hyperglycemia after treatment with dexamethasone for 3 weeks compared to control mice. Blood glucose raised less in glucose and pyruvate tolerance tests and dexamethasone-treated E47 knockout mice showed downregulation of GR target genes involved in gluconeogenesis in the liver (17).

In our study, we did not observe a correlation with fasting glucose concentrations, or with insulin or HOMA index or HbA1c.

As hyperglycemia in CS is caused not only by GC effects on the liver but also by a combination of GC effects on the liver, muscle, adipose tissue, and pancreas, and because we could not investigate hepatic E47 expression in human liver tissue, it might be difficult to detect a modulation of glucose concentrations by E47 *in vivo*.

The altered lipid metabolism and hyperglycemia observed in CS also contribute to the development of obesity (24). In particular, patients suffer from central obesity and show changes in body composition with a redistribution of body fat, increased abdominal visceral adipose tissue, reduced peripheral subcutaneous adipose fat deposition (25), and a characteristic decrease in muscle mass, resulting in thinning of arms and limbs (26). BMI, therefore, is not a sensitive marker to distinguish differences in body composition caused by CS. As a sensitive parameter for visceral obesity, we therefore investigated waist–arm ratio. We could show that E47 mRNA expression in blood correlated positively with waist–arm ratio in patients with CS in remission.

The limitations of our study are that we only included female patients due to a distinct female preponderance and a very small male cohort to perform sex-specific statistical analysis. The cohort might also have been too small to detect more subtle differences in clinical outcome markers, e.g., blood pressure, body composition, or insulin resistance. Strengths include the well-characterized patient cohort with extensive clinical data as well as a healthy control cohort with ACTH stimulation and Dex suppression testing and consistent results across groups. The development of therapeutics leading to E47 gene expression could have wide implications for multiple diseases across specialties that use chronic glucocorticoids.

To sum up, our data offer new insights into the regulation of GC-induced adverse effects. In particular, they provide a deeper understanding of the complex mechanisms of the pathophysiology of GC-induced dyslipidemia. We could show for the first time that the previously described findings in mice are also relevant to human physiology. E47 acts as a GC-dependent gene that is downregulated in glucocorticoid excess, potentially offering protection from glucocorticoid-induced comorbidities mediated by the liver.

## Data availability statement

The original contributions presented in the study are included in the article, further inquiries can be directed to the corresponding author/s.

## Ethics statement

The studies involving humans were approved by ehtics committee of the LMU Klinikum Munich, ethical approval no. 152-10. The studies were conducted in accordance with the local legislation and institutional requirements. The participants provided their written informed consent to participate in this study.

## Author contributions

WZ: literature search, figures, data collection, experimental work, data analysis, writing-original draft. HN: data analysis, data interpretation, writing - review and editing. MT: data interpretation, writing - review and editing JS: data analysis, writing - review and editing. CH: experimental work, data analysis, writing -review and editing. MB: data collection, resources, writing -review and editing. MR: data collection, data interpretation, writing - review and editing. HU: study design, methodology, data analysis, data interpretation, supervision, writing - review and editing. NR: study design, resources, methodology, supervision, data analysis, data interpretation, writing - original draft. All authors contributed to the article and approved the submitted version.

## References

[1] Pivonello R De Martino MC De Leo M Simeoli C Colao A. Cushing's disease: the burden of illness. Endocrine (2017) 56:10–8. doi: 10.1007/s12020-016-0984-8 27189147

[2] Shibli-Rahhal A Van Beek M Schlechte JA. Cushing's syndrome. Clin Dermatol (2006) 24:260–5. doi: 10.1016/j.clindermatol.2006.04.012 16828407

[3] Valassi E Santos A Yaneva M Toth M Strasburger CJ Chanson P. The European Registry on Cushing's syndrome: 2-year experience. Baseline demographic and clinical characteristics. Eur J Endocrinol (2011) 165:383–92. doi: 10.1530/EJE-11-0272 21715416

[4] Feelders RA Pulgar SJ Kempel A Pereira AM. The burden of Cushing's disease: clinical and health-related quality of life aspects. Eur J Endocrinol (2012) 167:311–26. doi: 10.1530/EJE-11-1095 22728347

[5] Friedman TC Mastorakos G Newman TD Mullen NM Horton EG Costello R. Carbohydrate and lipid metabolism in endogenous hypercortisolism: shared features with metabolic syndrome X and NIDDM. Endocr J (1996) 43:645–55. doi: 10.1507/endocrj.43.645 9075604

[6] Rockall AG Sohaib SA Evans D Kaltsas G Isidori AM Monson JP. Hepatic steatosis in Cushing's syndrome: a radiological assessment using computed tomography. Eur J Endocrinol (2003) 149:543–8. doi: 10.1530/eje.0.1490543 14640995

[7] Ramamoorthy S Cidlowski JA. Corticosteroids: mechanisms of action in health and disease. Rheum Dis Clin North Am (2016) 42:15–31. doi: 10.1016/j.rdc.2015.08.002 26611548 PMC4662771

[8] Oakley RH Cidlowski JA. The biology of the glucocorticoid receptor: new signaling mechanisms in health and disease. J Allergy Clin Immunol (2013) 132:1033–44. doi: 10.1016/j.jaci.2013.09.007 PMC4084612 24084075

[9] Cain DW Cidlowski JA. Specificity and sensitivity of glucocorticoid signaling in health and disease. Best Pract Res Clin Endocrinol Metab (2015) 29:545–56. doi: 10.1016/j.beem.2015.04.007 PMC4549805 26303082

[10] Miranda TB Morris SA Hager GL. Complex genomic interactions in the dynamic regulation of transcription by the glucocorticoid receptor. Mol Cell Endocrinol (2013) 380:16–24. doi: 10.1016/j.mce.2013.03.002 23499945 PMC3724757

[11] John S Sabo PJ Thurman RE Sung MH Biddie SC Johnson TA. Chromatin accessibility pre-determines glucocorticoid receptor binding patterns. Nat Genet (2011) 43:264–8. doi: 10.1038/ng.759 PMC6386452 21258342

[12] Granner DK Wang JC Yamamoto KR. Regulatory actions of glucocorticoid hormones: from organisms to mechanisms. Adv Exp Med Biol (2015) 872:3–31. doi: 10.1007/978-1-4939-2895-8_1 26215988

[13] Lim HW Uhlenhaut NH Rauch A Weiner J Hubner S Hubner N. Genomic redistribution of GR monomers and dimers mediates transcriptional response to exogenous glucocorticoid. vivo. Genome Res (2015) 25:836–44. doi: 10.1101/gr.188581.114 PMC4448680 25957148

[14] Bain G Robanus Maandag EC te Riele HP Feeney AJ Sheehy A Schlissel M. Both E12 and E47 allow commitment to the B cell lineage. Immunity (1997) 6:145–54. doi: 10.1016/s1074-7613(00)80421-5 9047236

[15] Teachenor R Beck K Wright LY Shen Z Briggs SP Murre C. Biochemical and phosphoproteomic analysis of the helix-loop-helix protein E47. Mol Cell Biol (2012) 32:1671–82. doi: 10.1128/MCB.06452-11 PMC3347234 22354994

[16] Schwartz R Engel I Fallahi-Sichani M Petrie HT Murre C. Gene expression patterns define novel roles for E47 in cell cycle progression, cytokine-mediated signaling, and T lineage development. Proc Natl Acad Sci USA (2006) 103:9976–81. doi: 10.1073/pnas.0603728103 PMC1502564 16782810

[17] Hemmer MC Wierer M Schachtrup K Downes M Hubner N Evans RM. E47 modulates hepatic glucocorticoid action. Nat Commun (2019) 10:306. doi: 10.1038/s41467-018-08196-5 30659202 PMC6338785

[18] Nieman LK Biller BM Findling JW Newell-Price J Savage MO Stewart PM. The diagnosis of Cushing's syndrome: an Endocrine Society Clinical Practice Guideline. J Clin Endocrinol Metab (2008) 93:1526–40. doi: 10.1210/jc.2008-0125 PMC2386281 18334580

[19] Nieman LK Biller BM Findling JW Murad MH Newell-Price J Savage MO. Treatment of Cushing's syndrome: an endocrine society clinical practice guideline. J Clin Endocrinol Metab (2015) 100:2807–31. doi: 10.1210/jc.2015-1818 PMC4525003 26222757

[20] Fassnacht M Tsagarakis S Terzolo M Tabarin A Sahdev A Newell-Price J. European Society of Endocrinology clinical practice guidelines on the management of adrenal incidentalomas, in collaboration with the European Network for the Study of Adrenal Tumors. Eur J Endocrinol (2023) 189:G1–G42. doi: 10.1093/ejendo/lvad066 37318239

[21] Berr CM Stieg MR Deutschbein T Quinkler M Schmidmaier R Osswald A. Persistence of myopathy in Cushing's syndrome: evaluation of the German Cushing's Registry. Eur J Endocrinol (2017) 176:737–46. doi: 10.1530/EJE-16-0689 28325824

[22] Braun LT Vogel F Rubinstein G Zopp S Nowak E Constantinescu G. Lack of sensitivity of diagnostic Cushing-scores in Germany: a multicenter validation. Eur J Endocrinol (2023) 188(1). doi: 10.1093/ejendo/lvac016 36651158

[23] Braun LT Zopp S Vogel F Honegger J Rubinstein G Schilbach K. Signs, symptoms and biochemistry in recurrent Cushing disease: a prospective pilot study. Endocrine (2021) 73:762–6. doi: 10.1007/s12020-021-02719-9 PMC8325659 33871792

[24] Luijten IHN Brooks K Boulet N Shabalina IG Jaiprakash A Carlsson B. Glucocorticoid-induced obesity develops independently of UCP1. Cell Rep (2019) 27:1686–1698 e1685. doi: 10.1016/j.celrep.2019.04.041 31067456

[25] Xiao X Li H Yang J Qi X Zu X Yang J. Wnt/beta-catenin signaling pathway and lipolysis enzymes participate in methylprednisolone induced fat differential distribution between subcutaneous and visceral adipose tissue. Steroids (2014) 84:30–5. doi: 10.1016/j.steroids.2014.03.004 24657224

[26] Makras P Toloumis G Papadogias D Kaltsas GA Besser M. The diagnosis and differential diagnosis of endogenous Cushing's syndrome. Hormones (Athens) (2006) 5:231–50. doi: 10.14310/horm.2002.11189 17178699

